# Tumor-intrinsic IFNα and CXCL10 are critical for immunotherapeutic efficacy by recruiting and activating T lymphocytes in tumor microenvironment

**DOI:** 10.1007/s00262-024-03761-y

**Published:** 2024-07-02

**Authors:** Chun-Chia Cheng, Jungshan Chang, Ai-Sheng Ho, Zong-Lin Sie, Cheng-Liang Peng, Chih‑Liang Wang, Kapil Dev, Chun-Chao Chang

**Affiliations:** 1 Research Center of Radiation Medicine, Chang Gung University, Taoyuan, 333 Taiwan; 2 Division of Pulmonary Oncology and Interventional Bronchoscopy, Department of Thoracic Medicine, Chang Gung Memorial Hospital, Linkou, Taoyuan, 333 Taiwan; 3 Graduate Institute of Medical Sciences, School of Medicine, College of Medicine, Taipei Medical University, Taipei, 110 Taiwan; 4 Division of Gastroenterology, Cheng Hsin General Hospital, Taipei, 112 Taiwan; 5 Department of Isotope Application Research, National Atomic Research Institute, Taoyuan, 325 Taiwan; 6 Division of Gastroenterology and Hepatology, Department of Internal Medicine, Taipei Medical University Hospital, Taipei, 110 Taiwan; 7 Division of Gastroenterology and Hepatology, Department of Internal Medicine, School of Medicine, College of Medicine, Taipei Medical University, Taipei, 110 Taiwan; 8 TMU Research Center for Digestive Medicine, Taipei Medical University, Taipei, 110 Taiwan

**Keywords:** IFNα, CXCL10, PD-L1, Immunotherapy, Lung cancer, Colon cancer, LL/2, CT26

## Abstract

Tumor immunotherapies targeting PD-(L)1 exhibit anti-tumor efficacy in only 10–30% of patients with various cancers. Literature has demonstrated that a “hot tumor” which contains high T lymphocytes in the tumor microenvironment exhibits a better response to immunotherapies than a “cold tumor.” This study aimed to investigate whether tumor-intrinsic IFNα and CXCL10 determine the recruitment and activation of CD8^+^ T cells to become “hot tumor.” In this study, we found that CXCL10 overexpressed in a variety of tumors including lung, colon, and liver tumors with a correlation with PD-L1. High PD-L1 and CXCL10 are associated with better survival rates in tumor patients receiving immunotherapies. IFNs-downstream transcriptional factor IRF-1 and STAT1 were correlated with PD-L1 and CXCL10 expression. We demonstrated that IRF-1 and STAT1 were both bound with the promoters of PD-L1 and CXCL10, sharing the same signaling pathway and determining IFNs-mediated PD-L1 and CXCL10 expression. In addition, IFNα significantly increased activation marker IFNγ in PBMCs, promoting M1 type monocyte differentiation, CD4^+^ T, and CD8^+^ T cell activation. Particularly, we found that CD8^+^ T lymphocytes abundantly expressed CXCR3, a receptor of CXCL10, by flow cytometry, indicating that tumor-intrinsic CXCL10 potentially recruited CD8^+^ T in tumor microenvironment. To demonstrate the hypothesis, immunotherapy-sensitive CT26 and immunotherapy-resistant LL/2 were used and we found that CT26 cells exhibited higher IFNα, IFNγ, CXCL10, and PD-L1 levels compared to LL/2, leading to higher IFNγ expression in mouse splenocytes. Moreover, we found that CD8^+^ T cells were recruited by CXCL10 in vitro, whereas SCH546738, an inhibitor of CXCR3, inhibited T cell migration and splenocytes-mediated anti-tumor effect. We then confirmed that CT26-derived tumor was sensitive to αPD-L1 immunotherapy and LL/2-tumor was resistant, whereas αPD-L1 significantly increased T lymphocyte activation marker CD107a in CT26-derived BALB/c mice. In conclusion, this study revealed that CXCL10 expression is correlated with PD-L1 in tumors, sharing the same signaling pathway and associating with better immunotherapeutic efficacy. Further evidence in the syngeneic tumor models demonstrated that immunotherapy-sensitive CT26 intrinsically exhibited higher IFNα and CXCL10 compared to immunotherapy-resistant LL/2 to recruit and activate CD8^+^ T cells in the tumor microenvironment, exhibiting “hot tumor” characteristic of sensitizing αPD-L1 immunotherapies.

## Introduction

Mutations and amplifications of oncogenes contribute to cancer cell uncontrolled proliferation and survival. Suppressing tumor cells and prolonging patient survival rate are critical in clinical tumor treatments. To date, immunotherapies are promising to inhibit cancer cell proliferation by targeting the immune checkpoints, such as PD-(L)1. Since lung cancer, liver cancer, and colon cancer all exhibit PD-L1 overexpression [1–4], immunotherapies targeting PD-(L)1 are considered potential in treating such types. However, there have only been 10–30% of patients receiving immunotherapies that result in good response [5]. It is critical to investigate the mechanism and to enhance the immunotherapeutic efficacy in clinical practice.

Tumors have developed the overexpression of inhibitory signal PD-L1 to exhaust CD8^+^ T cells in patients with cancers [6]. Immunotherapies blocking PD-1-to-PD-L1 interaction have potential to reactivate CD8^+^ T cells, which are well-known as cytotoxic T lymphocytes (CTLs) for killing the aberrant tumor cells [7]. αPD-(L)1 immunotherapies have demonstrated promising efficacies in lung cancer and hepatocellular carcinoma (HCC). For example, Nivolumab, a PD-1 antibody, was approved in 2015 to treat patients with lung cancer after platinum doublet chemotherapy failed [8]; for patients with HCC, Nivolumab is approved for tumor treatment based on the results from the CheckMate 040 trial [9]. Currently, Nivolumab is evaluated as a first-line therapy for advanced HCC in the CheckMate 459 clinical trial [10]. For advanced CRC, αPD-(L)1 immunotherapies are potential and available [11]. Currently, 10–30% of patients with good immunotherapeutic response are considerably “hot tumor,” who have T cell infiltration and present neoantigens in tumors to be recognized by CD8^+^ T cells [12]. To enhance immunotherapeutic efficacy, by transferring “cold tumor” to “hot tumor” is considered a potential strategy.

Literature has indicated that a high density of CTLs in tumor tissue is usually beneficial for patients and correlated with a better therapeutic outcome [13–15]. Because “cold tumors” are characterized by the absence of immune cells or the limited number of cytotoxic immune cells in the tumor microenvironment. Therefore, “cold tumors” do not respond to immunotherapy. Treatment of cold tumors with some inhibitors such as Vps34 inhibitors can induce the release of inflammatory chemokines such as CCL5 and CXCL10 to recruit natural killer (NK) and CD8^+^ T cells to the tumor microenvironment, resulting in a better immunotherapeutic efficacy [16]. Previous studies have demonstrated that low doses of fractionated radiotherapy (RT) also significantly improve CD8^+^ T cell-mediated tumor remission, particularly in combination with anti-PD-1 or anti-PD-L1 therapies [17–19]. Therefore, RT-mediated tumor therapies are considered for improving the anti-tumor efficacy of clinical immunotherapies [20–22] by inducing tumor IFNα and CXCL10 expression [23]. We have noticed that CT26 exhibits αPD-(L)1 immunotherapy-sensitive but LL/2 exhibits immunotherapy-resistant [24, 25]. Therefore, we proposed and investigated whether LL/2 presented “cold tumor” characteristics, and CT26 presented “hot tumor” characteristics with differential IFNα and CXCL10 levels to determine the immunotherapeutic efficacy in this study.

## Materials and methods

### Bioinformatic analysis

The Gene Expression Profiling Interactive Analysis (GEPIA, http://gepia.cancer-pku.cn/) was used to analyze the gene expression for patients with lung cancer (LUAD), liver cancer (LIHC), and colorectal cancer (COAD) based on the publicly available databases such as The Cancer Genome Atlas (TCGA). In addition, cBioPortal (https://www.cbioportal.org/) was used to analyze the gene correlation, including PD-L1, ICAM-1, CXCL10, IRF1, and STAT1. Kaplan–Meier plotter (https://kmplot.com/analysis/) was used to analyze the correlation between the mRNA expression and survival probability, and the correlation between genes and anti-PD-1 (PD-L1) immunotherapeutic survival rate in patients with all tumors, lung cancer (non-small cell lung cancer, NSCLC), and liver cancer (hepatocellular carcinoma, HCC) based on gene expression omnibus (GEO), European Genome-phenome Archive (EGA), and TCGA databases.

### Cell culture

The human cancer cell line (A549, PLC5, HT29, Jurkat) and mouse cancer cell lines (Hepa1-6, LL/2, CT26) used in this study were purchased from the American Type Culture Collection (ATCC, Manassas, VA, USA). All the cell lines were reauthenticated through short tandem repeat profiling (Applied Biosystems, Waltham, MA, USA) before this study. Jurkat was cultured in Roswell Park Memorial Institute (RPMI) medium, and other cell lines were cultured in Dulbecco’s Modified Eagle’s Medium (DMEM). All the culture medium was supplied with 10% fetal bovine serum (FBS) and 1% penicillin–streptomycin (P/S). All the cells were cultured at 37 °C with 5% CO_2_.

### Animal

Male C57BL/6 mice and BALB/C mice were purchased from the National Laboratory Animal Center, Taiwan. The mice were housed in a 12 h-light cycle at 22 °C. The animal studies were approved by the ethical review committee in Chan Gung University, Taiwan (CGU110-164), which followed the NIH guidelines on the care and welfare of laboratory animals. Syngeneic tumor graft models were established by subcutaneously injecting 1 × 10^6^ LL/2 cells into the legs of C57BL/6 mice and 1 × 10^6^ CT26 cells into the legs of BALB/c mice of 3-month-old. After 1-week post-injection, tumor-bearing mice (*n* = 3) were treated with 3 doses of 100 µg αPD-L1scFv-hFc by intraperitoneal injection every 2 days. Tumor volume was recorded and calculated using the formula: 0.52 × width^2^ × length, herein the width represents the smaller tumor diameter. In addition, the body weight was recorded as a toxicity marker in αPD-L1scFv-hFc administration.

### Chromatin immunoprecipitation (ChIP)

In brief, 1 × 10^7^ A549 cells were treated with 20 ng/mL of IFNα/γ mixture for 2 h. After treatment, A549 cells were fixed by 1% formaldehyde and fragmentized by sonication. A549 lysates were resuspended and incubated with 2 μg of control IgG, anti-IRF1, and anti-STAT1 antibodies (Cell Signaling, Danvers, Massachusetts, USA) at 4 °C overnight for immunoprecipitation. After incubation, the antibodies were captured by 0.25 mg of Dynabeads-Protein A (Life Technologies, Waltham, Massachusetts, USA). The Dynabeads were washed and consequently pulled down by a Sample Magnetic Rack. DNA fragments were eluted by boiling the Dynabeads and concentrated by a FavorPrep GEL/PCR Purification Mini Kit (Favorgen Biotech Corp., Wembley, WA, Australia). The DNA preparation was analyzed by real-time PCR using a Fast SYBR Green Master Mix (Applied Biosystem, CA, USA) with primer pairs shown in Table 1. The primer sequences amplify the PD-L1 and CXCL10 promoter predicted from PROMO (http://alggen.lsi.upc.es/).

**Table 1:** Human qPCR primers

Gene	Direct	Primer sequence
*IRF1*	Forward	AGCTCAGCTGTGCGAGTGTA
	Reverse	TAGCTGCTGTGGTCATCAGG
*STAT1*	Forward	CCGTTTTCATGACCTCCTGT
	Reverse	TGAATATTCCCCGACTGAGC
*CD274 *(PD-L1)	Forward	GTACCTTGGCTTTGCCACAT
	Reverse	CCAACACCACAAGGAGGAGT
*CXCL10*	Forward	CTGTACGCTGTACCTGCATCA
	Reverse	TTCTTGATGGCCTTCGATTC
*ISG15*	Forward	TGTCGGTGTCAGAGCTGAAG
	Reverse	GCCCTTGTTATTCCTCACCA
*CD38*	Forward	TTGGGAACTCAGACCGTACC
	Reverse	GTTGCTGCAGTCCTTTCTCC
*NOS2 *(iNOS)	Forward	ACAAGCCTACCCCTCCAGAT
	Reverse	TCCCGTCAGTTGGTAGGTTC
*TNFA*	Forward	CAGAGGGCCTGTACCTCATC
	Reverse	GGAAGACCCCTCCCAGATAG
*CCL2*	Forward	CCCCAGTCACCTGCTGTTAT
	Reverse	TGGAATCCTGAACCCACTTC
*CD206*	Forward	GGCGGTGACCTCACAAGTAT
	Reverse	ACGAAGCCATTTGGTAAACG
*ARG1*	Forward	GGCTGGTCTGCTTGAGAAAC
	Reverse	ATTGCCAAACTGTGGTCTCC
*VEGFA*	Forward	CCCACTGAGGAGTCCAACAT
	Reverse	TTTCTTGCGCTTTCGTTTTT
*IL-10*	Forward	GCCAAGCCTTGTCTGAGATG
	Reverse	AAGAAATCGATGACAGCGCC
*IFNG*	Forward	TCCCATGGGTTGTGTGTTTA
	Reverse	AAGCACCAGGCATGAAATCT
*IL-2*	Forward	TGCAACTCCTGTCTTGCATT
	Reverse	GCCTTCTTGGGCATGTAAAA
*GZMB*	Forward	ATGCAACCAATCCTGCTTCT
	Reverse	CCCCAAGGTGACATTTATGG
*PRF1*	Forward	ACTCACAGGCAGCCAACTTT
	Reverse	GGGTGCCGTAGTTGGAGATA
*BCL2*	Forward	GAGGATTGTGGCCTTCTTTG
	Reverse	ACAGTTCCACAAAGGCATCC
*GAPDH*	Forward	GAGTCAACGGATTTGGTCGT
	Reverse	TTGATTTTGGAGGGATCTCG
ChIP-*PDL1*	Forward	TGAACTTCCAATTCCCTGTTG
	Reverse	TCATCTTTCTGGAATGCCCTA
ChIP-*CXCL10*	Forward	TCATGTTTTGGAAAGTGAAACC
	Reverse	CCCTCCCTAATTCTGATTGGA

### Quantitative polymerase chain reaction (qPCR)

Total RNA from tumor cells and PBMCs with indicated treatments was isolated using TRIzol reagent (Thermo Fisher Scientific, Waltham, MA, USA). In brief, cells were lysed in 500 μL TRIzol mixed with 100 μL of 1-bromo-3-chloropropane. After 13,000-rpm centrifugation for 15 min at 4 °C, RNA remained in 250 μL of the aqueous phase and was transferred to a fresh tube and precipitated by adding 250 μL of isopropanol. The pellet was collected after 13,000-rpm centrifugation for 30 min at 4 °C and washed with 300 μL of 70% ethanol. Furthermore, the pellet was dissolved in RNase-free water after air drying and the concentration was subsequently detected using a SpectraMax iD3 Multi-Mode Microplate Reader (Molecular Devices, San Jose, CA, USA). Consequently, the complementary DNA was synthesized from 1 μg of RNA using a High-Capacity cDNA Reverse Transcription Kit (Thermo Fisher Scientific, Waltham, MA, USA) according to the manufacturer’s protocol. qPCR was performed using a SYBR Green-based system (Thermo Fisher Scientific, Waltham, MA, USA). The expression of specific genes was quantified based on a 3-times repeat with normalization to GAPDH. The primer sequences are listed in Tables 1 and 2.

**Table 2:** Mouse qPCR primers

Gene	Direct	Primer sequence
*Ifna*	Forward	CAGCAGCTCAATGACCT
	Reverse	GGCTGTGTTTCTTCTCTCTC
*Ifng*	Forward	ACTGGCAAAAGGATGGTGAC
	Reverse	TGAGCTCATTGAATGCTTGG
*Cxcl10*	Forward	AAGTGCTGCCGTCATTTTCT
	Reverse	GTGGCAATGATCTCAACACG
*Pd-l1*	Forward	CTGCCAAAGGACCAGCTTTT
	Reverse	GGCTGGATCCACGGAAATTC
*Icam1*	Forward	TTCACACTGAATGCCAGCTC
	Reverse	GTCTGCTGAGACCCCTCTTG
*Il-2*	Forward	GCGGCATGTTCTGGATTTGACTC
	Reverse	CCACCACAGTTGCTGACTCATC
*Gapdh*	Forward	ACTCCACTCACGGCAAATTC
	Reverse	TCTCCATGGTGGTGAAGACA

### PCR to validate the gene expression in CT26 and LL/2

To verify the expression of Ifna and Cxcl10 in CT26 and LL/2 cells, the Ifna and Cxcl10 primers (Table 2) were used to amplify 127-bp and 186-bp fragments from the cDNA of CT26 and LL/2 using a twofold pfu PCR mix (Bioman, New Taipei City, Taiwan). The PCR assays contained 0.5 μL of cDNA (25 ng), 10 μL of 2× pfu PCR mix, and 20 pmoL of each primer. The PCR cycle started with an initial denaturation at 95 °C for 2 min; afterward, cycling conditions were 35 cycles of 95 °C for 30 min, 55 °C for 30 min, and 72 °C for 1 min, followed by a final elongation step at 72 °C for 10 min. PCR amplifications were performed with a BioRad T100 thermal cycler (Biorad, California, USA). The PCR products were analyzed by electrophoresis in 1% agarose gel and onefold TAE buffer (40 mM Tris–acetate, 1 mM EDTA) at 110 V for 30 min.

### Flow cytometry

For flow cytometry analysis, individual 1 × 10^6^ cells were in 100 μL of 0.1% FBS-PBS and incubated with indicated antibodies (1:100 dilute, BioLegend, San Diego, CA, USA) for 30 min on ice. After incubation, cells were washed and resuspended in 1 mL of 0.1% FBS-PBS. The cells were then analyzed using an Attune NxT Flow Cytometer (Invitrogen, Waltham, MA, USA). The anti-human CD274-PE antibodies were used to detect PD-L1 expression in A549, and anti-mouse CD274-PE for Hepa1-6, LL/2, and CT26 cells. The anti-CD183-Alexa700 antibodies were used to detect CXCR3 expression in the A549, HT29, PLC5, and Jurkat cells. Moreover, CXCR3 expression in CD4^+^/CD8^+^ T cells from human PBMCs and mouse C57BL6 splenocytes was also detected using the same method. PBMCs were stained with anti-CD45-Pacific blue, anti-CD3-APC/Cy7, anti-CD8-Alexa488, anti-CD4-PE/Cy7, and anti-CD183-Alexa700 or anti-IFNγ-PE. Splenocytes were stained with anti-CD45-PerCP/Cy5, anti-CD3-Pacific blue, anti-CD8-Alexa700, and anti-CD183-PE.

Moreover, to detect the binding capacity of αPD-L1scFv-hFc, 1 × 10^6^ LL/2 and CT26 cells were resuspended in 100 μL of 0.1% FBS-PBS and incubated with 2 μg/mL of αPD-L1scFv-hFc on ice for 1 h. Afterward, cells were washed with 1 mL of 0.1% FBS-PBS and sequentially incubated with 2 μg/mL of Rabbit anti-His (ABclonal, Woburn, MA, USA) and Alexa647 Goat anti-Rabbit IgG (Elabscience, Houston, Texas, USA) antibodies on ice for 30 min. After incubation, cells were washed and resuspended in 1 mL of 0.1% FBS-PBS. The cells were then analyzed using an Attune NxT Flow Cytometer (Invitrogen, Waltham, MA, USA).

### Isolation of splenocytes

Spleen was harvested and transferred to a Petri dish containing 5 mL of ice-cold RPMI medium. The tissues were homogenized by gently pressing the spleen through a 40 µm cell strainer into the Petri dish with a flat plunger end of a syringe. The splenocyte suspension was transferred to a 15-mL centrifuge tube and sedimented at 1200 rpm for 10 min at 4 °C. Erythrocytes were lysed with 1 mL of RBC lysis buffer (Sigma-Aldrich, St. Louis, MO, USA). After PBS wash, the splenocytes were resuspended in RPMI culture media for future assays.

### Lentivirus-mediated gene knockdown and overexpression

Gene knockdown and gene overexpression were conducted using lentivirus expression vectors which were purchased from the National RNAi Core Facility of Academia Sinica, Taipei, Taiwan. For gene knockdown, the specific short hairpin RNAs (shRNA) were inserted into the pLKO.1-puro vector for gene knockdown, whereas shLuciferase (shLuc) was used as a negative control. For gene overexpression, the mouse Cxcl10 gene was inserted into the pLAS3w.Puro vector, whereas pLAS3w-RFP was used as a negative control.

To generate lentivirus, 293 T cells in a 6-well plate by 70% confluence were transfected with 1 μg of the pLKO.1 or pLAS3w vector, 0.25 μg of the envelope plasmid pVSV-G, and 0.9 μg of the packaging plasmid pCMVΔR8.91. The plasmids were preincubated with 6 μL of Maestrofectin Transfection Reagent (Omics Bio, New Taipei, ROC) and 50 μL of serum-free DMEM for 30 min at room temperature and subsequently added to the 293 T cells. The culture medium was replaced with a fresh culture medium with 30% FBS after 24 h and incubated for another 48 h to generate the virus. The cultured medium containing virus was collected and stored at − 80 °C. For virus infection, A549 or CT26 cells cultured by 80% confluence were infected with the prepared lentivirus (preincubated with 8 µg/mL of polybrene) for 24 h. The cells were then transferred to a DMEM medium containing 4 µg/mL of puromycin and harvested after stable cells were obtained.

### Cxcl10-pLAS3w.Puro plasmid construction

The mouse Cxcl10 gene was amplified from splenocyte cDNA using a twofold pfu PCR mix (Bioman, New Taipei City, Taiwan) with following primer pairs: forward primer containing NheI site: 5′-ATCGGCTAGCCATGGGCAACCCAAGTGC-3′; reverse primer containing EcoRI site: 5′-TAGCGAATTCAAGGAGCCCTTTTAGACCTTTTTTGGC-3′. The PCR cycle started with an initial denaturation at 95 °C for 2 min; afterward, cycling conditions were 35 cycles of 95 °C for 30 min, 55 °C for 30 min, and 72 °C for 1 min, followed by a final elongation step at 72 °C for 10 min. PCR amplifications were performed with a BioRad T100 thermal cycler (Biorad, California, USA). The amplified Cxcl10 was sub-cloned into the multiple cloning site of the pLAS3w vector by restriction digestion with NheI and EcoRI, followed by ligation (New England Biolabs, Ipswich, Massachusetts, USA).

### Isolation of peripheral blood mononuclear cells (PBMCs)

The human study was approved by the regulatory authorities and Institutional Review Boards at Chang Gung Memorial Hospital, Linkou, Taiwan (202102475B0, March 3, 2022). Signed and informed written consent was obtained from all participants, and the research was performed under the relevant guidelines and regulations. PBMCs from a healthy volunteer were isolated via density gradient centrifugation over Ficoll-Paque PLUS media (Cytiva, Marlborough, MA, US). In brief, 10 mL of whole blood were centrifuged at 1200 rpm for 30 min at 20 °C without braking. After centrifuge, 3 mL of buffy coats were collected and mixed with 4 mL of PBS buffer. The diluted buffy coats were generally loaded on 4 ml of Ficoll media and consequently centrifuged at 2000 rpm for 30 min at 20 °C without braking to generate distinct layers of plasma, PBMC, Ficoll media, and red blood cell (RBC). PBMCs were harvested and remained erythrocytes in the PBMCs were lysed with 1 mL of RBC lysis buffer (Sigma-Aldrich, St. Louis, MO, USA). After PBS wash, the PBMCs were resuspended in RPMI culture media for future assays.

### CD8^+^ T cell migration in vitro

A transwell migration assay (3.0 μm) was used to detect CD8^+^ T cell migration capacity (JET biofil, JET biofil, China). In brief, 1 × 10^4^ CT26-RFP and CT26-Cxcl10 cells in 750 μL of RPMI culture medium were treated with 0.1 μM of SCH546738 (CXCR3 antagonist) in a 24-well culture plate. Then, 1 × 10^6^ splenocytes labeled with PE-mCD8 antibody (BioLegend, San Diego, CA, USA) were placed in the upper layer of a cell culture insert with 150 μL of RPMI culture medium. Cells were incubated at 37 °C and 5% CO_2_ for 24 h. The migrated cells that had through the membrane filter were counted in 4 independent views under a microscope.

### Isolation of CD4^+^, CD8^+^ T cells, and nonT PBMCs

Following the manufacturer’s manual the CD4^+^, CD8^+^ T cells, and nonT PBMCs were isolated using MACS Cell Isolation Kits, human (Miltenyi Biotec, North Rhine-Westphalia, Germany). In brief, individual 5 × 10^6^ PBMCs were labeled with 10 μL of CD8 magnetic microbeads for 15 min at 4 °C. After incubation, PBMCs were resuspended in 0.5 mL of MACS buffer (1% FBS and 1 mM EDTA in PBS) and applied to the top of MASC LS Columns placed in a Magnetic Separator. The column was washed with 1 mL of MACS buffer for 3 times to flush out unbound cells. Consequently, the column was removed from the Magnetic Separator, and CD8^+^ T retained in the column was harvested by vigorously flushing using a plastic plunger with 1 mL of MACS buffer. Afterward, CD4^+^ T cells were isolated from the unbound cells using CD4 magnetic microbeads, which followed the same procedure described above. After CD8^+^ T and CD4^+^ T separation, the remaining cells were collected as nonT PBMCs, which theoretically contain dendritic cells (DCs), macrophages, and natural killer cells (NK).

### Construction and purification of mCxcl10-hFc and αPD-L1scFv-hFc recombinant protein

The mouse Cxcl10 gene was amplified from Cxcl10-pLAS3w.Puro plasmid by using a twofold pfu PCR mix (Bioman, New Taipei City, Taiwan) with the following primer pairs: forward primer containing NcoI site: 5′-ATCGCCATGGGCAACCCAAGTGCTGCCG-3′; reverse primer containing NheI site: 5′-TAGCGCTAGCAGGAGCCCTTTTAGACCTTTTTT-3′, and sub-cloned into pET28a-hFc vector. The pET28a-hFc encoding wild-type human IgG1 heavy chain (hFc) was synthesized by Synbio Technologies (Monmouth Junction, New Jersey, USA). Moreover, the pET28a-αPD-L1scFv-hFc encoding the single-chain variable fragment (scFv) from Atezolizumab was also synthesized by Synbio Technologies. The *Escherichia coli* strains BL21 (DE3) were used for protein induction and expression. The recombinant strains were grown in terrific broth in the presence of kanamycin (25 μg/mL) as appropriate at 37 °C. When the culture had reached an optical density (OD 600) of 0.5, isopropyl-β-d-thiogalactopyronoside (IPTG) at 1 mM was added to induce protein expression and then growth was continued for 4 h. After IPTG induction, the *E. coli* cells were harvested using centrifugation for 30 min at 2000 rpm.

To purify the recombinant proteins, each gram of bacteria pellets was resuspended in 5 mL of binding buffer (50 mM NaH_2_PO_4_, 300 mM NaCl, 10 mM imidazole, pH = 8.0) with 1 mg/mL of lysozyme and then incubated at 4 °C for 30 min. The lysate was then sonicated on ice for 10 min and then incubated with 5 μg/mL of DNase I at 4 °C for 15 min. Afterward, the lysate was centrifuged at 10,000 rpm for 30 min at 4 °C to collect the inclusion bodies. The inclusion bodies were resuspended in 5 mL of denature binding buffer (50 mM NaH_2_PO_4_, 300 mM NaCl, 10 mM imidazole, 8 M urea, pH = 8.0) and incubated for 1 h at 4 °C. The solution was then centrifuged for 30 min at 10,000 rpm to remove the cell debris. The resulting cell supernatant was incubated with 2 mL of Nickel NTA Agarose Bulk Resins (Agarose Beads Technologies, Doral, Florida, USA) overnight at 4 °C. After incubation, the resin was packed into a plastic PD-10 column (Cytiva, Marlborough, MA, US). The packed column was washed with a tenfold volume of denature washing buffer (50  mM NaH_2_PO_4_, 300 mM NaCl, 20 mM imidazole, 8 M urea, pH 8.0) for 3 times, and the bound proteins were eluted with 4 mL of denature elution buffer (50 mM NaH_2_PO_4_, 300 mM NaCl, 250 mM imidazole, 8 M urea, pH = 8.0). The elution was dialyzed against onefold PBS (pH = 7.4) with 5 mg/mL sucrose and 1 mM EDTA, overnight at 4 °C. The total protein concentration of each fraction was determined using a BCA assay kit (Sigma-Aldrich, St. Louis, MO, USA) using bovine serum albumin as the reference protein. The purity of the protein sample was analyzed using aliquots of the fractions by 12.5% SDS-PAGE and ProteoSilver™ Silver Stain Kit (Sigma-Aldrich, St. Louis, MO, USA).

### Immunoprecipitation and detection of IFNα by Western blots

The supernatant from 1 × 10^6^ LL/2 and CT26 cells in a 10-cm dish was collected centrifuged at 2000 rpm for 10 min to remove cell debris. The supernatant was consequently incubated with 1 μg/mL of rabbit-anti-IFNα antibodies (Elabscience, Houston, Texas, USA) at 4 °C overnight. The antibodies were consequently captured by 0.2 mg/mL of Dynabeads-Protein A (Life Technologies, Waltham, Massachusetts, USA) at room temperature for 2 h. The Dynabeads were pulled down by a Sample Magnetic Rack, and the supernatant was removed. To make sure that IFNα was expressed in the medium, Western blots were used for detecting IFNα. In brief, Dynabeads were washed with 1 mL of PBS buffer and resuspended in 100 μL of RIPA buffer (50 mM Tris, 150 mM NaCl, 0.5% sodium deoxycholate, 1% IGEPAL, and 0.1% SDS) with 20 μL of sixfold SDS sample buffer (Alfa Aesar, Heysham, Lancashire, UK). The same antibodies targeting IFNα were used to detect the targets in Western blots.

### Statistical analysis

Statistical analyses were performed using GraphPad Prism V 8.01 (GraphPad Software, Inc., CA, USA). Significant differences for comparison of every two groups were assessed by unpaired two-tailed Student’s *t* test, whereas more groups were evaluated using ANOVA followed by Tukey’s or Sidak’s multiple comparison test. A *p* value less than 0.05 was considered statistically significant.

## Results

### PD-L1 and CXCL10 levels are both correlated with better immunotherapeutic efficacy

**Fig. 1:**
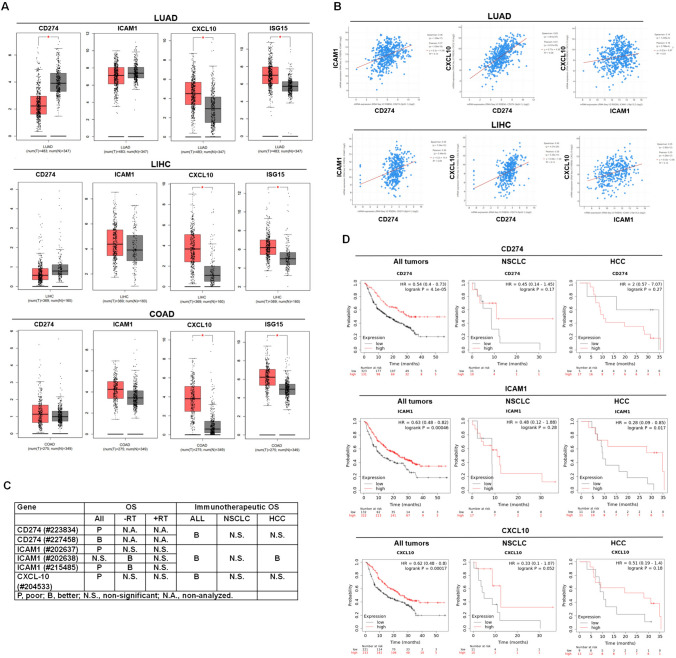
PD-L1 (CD274) and CXCL10 levels are correlated in cancer patients, both of which were also associated with better immunotherapeutic efficacy. **A** GEPIA was used to investigate the gene expression, including CD274, ICAM1, CXCL10, and IFNs-regulated ISG15, in patients with lung cancer, liver cancer (LIHC), and colorectal cancer (COAD) based on the TCGA database. Red: tumor (T); Gray: non-tumor (N). **B** cBioPortal was used to validate the correlation expression among CD274, ICAM1, and CXCL10 in LUAD and LIHC. **C** and **D** Kaplan–Meier plotter was used to investigate the association among CD274, ICAM1, and CXCL10 levels with immunotherapeutic efficacy in patients with all cancers (*n* = 454), non-small cell lung cancer (NSCLC, *n* = 21), and hepatocellular carcinoma (HCC, *n* = 22). OS, overall survival. *p < 0.05

PD-L1 expression in tumors is considered a marker to predict the immunotherapeutic efficacy in clinical practice. Overexpression of PD-L1 is just one of the essential factors determining the αPD-(L)1 immunotherapeutic efficacy. T lymphocytes and NK cells accumulation in tumor tissues are necessary and critical for immunotherapeutic anti-tumor efficacy [16], which is recruited by CXCL9, 10, and 11. Therefore, we investigated the expression of PD-L1, ICAM-1, CXCL10, and IFNs-mediated ISG15 expression in patients with lung cancer (LUAD), liver cancer (LIHC), and colorectal cancer (COAD) using the available GEPIA based on the TCGA database. The results indicated that CXCL10 and ISG15 were up-regulated in LUAD, LIHC, and COAD compared to the non-tumor group (Fig. 1A). However, PD-L1 exhibited down-regulation in LUAD and no differential in LIHC and COAD (Fig. 1A). The results were surprising since PD-L1 and CXCL10 were both considered regulated by IFNs, whereas IFNs-mediated ISG15 was a control to be compared with, which showed similar to CXCL10 expression (Fig. 1A). Further to demonstrate the expressional correlation among the selected genes, we found that PD-L1 and CXCL10 were correlated (*R*^2^ = 0.38 in LUAD and *R*^2^ = 0.14 in LIHC, Fig. 1B, whereas no COAD database in cBioPortal). We also found that high PD-L1, ICAM1, and CXCL10 levels all correlated with better immunotherapeutic efficacy in patients with all cancers (Fig. 1C, D). When specifically analyzing the association between the selected genes and immunotherapeutic efficacy in NSCLC and HCC, there was non-significant (except for ICAM1 in HCC) due to the limited sample number (*n* = 21 in NSCLC and *n* = 22 in HCC, Fig. 1C, D). Overall, PD-L1 and CXCL10 levels in tumor tissues may be correlated according to the TCGA database in cBioPortal. In addition, the high levels of PD-L1 and CXCL10 were associated with a better overall survival rate in all cancer patients who received immunotherapies (*n* = 454).

### IFNα/γ-downstream IRF1 and STAT1 mediate-PD-L1 and CXCL10 expression in A549 tumor cells

**Fig. 2:**
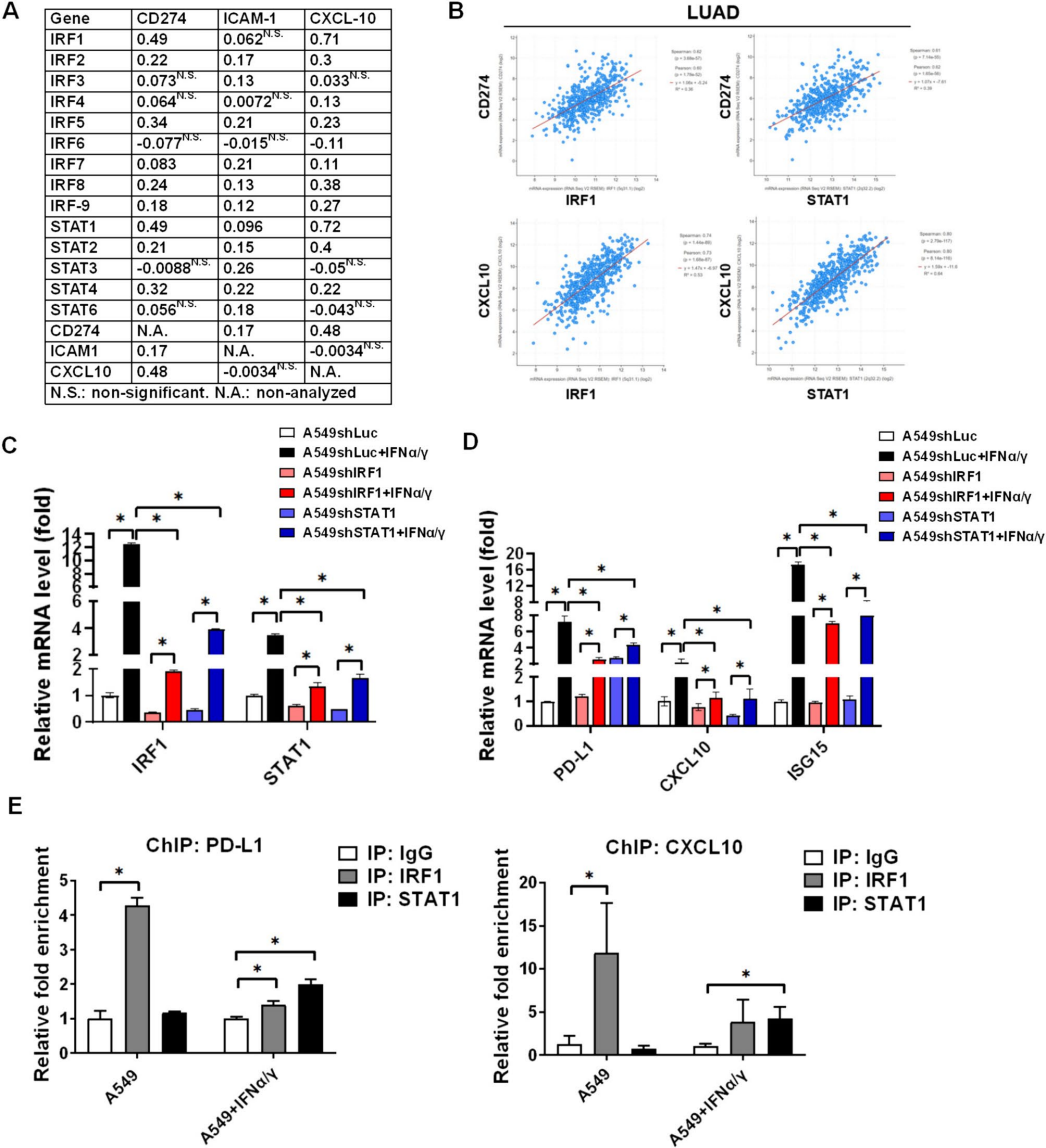
IRF1 and STAT1 mediate PD-L1 (CD274) and CXCL10 expression in tumor cells. **A** GEPIA based on the TCGA tumor database was used to investigate the correlation (*r*) between genes in patients with lung cancer adenocarcinoma, particularly the transcriptional factors IRFs and STATs with CD274, ICAM1, and CXCL10. **B** cBioPortal was used to further validate the correlation among IRF1, STAT1, CD274, and CXCL10 in patients with lung cancer adenocarcinoma (LUAD). (C) IRF1 and STAT1 were knocked down by shRNA and detected in 20 ng/mL of IFNα and IFNγ mixture treated A549 cells using qPCR. (D) PD-L1, CXCL10, and ISG15 were measured in 20 ng/mL of IFNα/γ mixture treated A549shLuc, A549shIRF1, and A549shSTAT1 tumor cells for 2 h using qPCR. (E) ChIP was used to investigate whether IRF1 and STAT1 were bound with the promoters of PD-L1 and CXCL10 in A549 cells. *p < 0.05

To investigate the potential mechanism determining PD-L1, ICAM-1, and CXCL10 expression in tumor cells, the levels of IFNα/γ-downstream transcriptional factors were used to correlate with PD-L1, ICAM-1, and CXCL10 expression by GEPIA and cBioPortal based on the TCGA database. We found that IRF1 and STAT1 were correlated with PD-L1 and CXCL10 (database in GEPIA, Fig. 2A). We validated that *R*^2^ = 0.36 between PD-L1 and IRF1; *R*^2^ = 0.53 between CXCL10 and IRF1; *R*^2^ = 0.39 between PD-L1 and STAT1; *R*^2^ = 0.64 between CXCL10 and STAT1 (database in cBioPortal, Fig. 2B). The results indicated that IFNα/γ-downstream IRF1 and STAT1 both potentially mediated PD-L1 and CXCL10 expression in lung cancer. Therefore, IRF1 and STAT1 were knockdown in lung cancer A549 cells (Fig. 2C), which decreased 20 ng/mL of IFNα/γ-mediated gene expression, including IRF1, STAT1 (Fig. 2C), PD-L1, CXCL10, and ISG15 (Fig. 2D). To validate that IRF1 mediates PD-L1 and CXCL10 expression, we demonstrated that IRF1 bound with the promoters of PD-L1 and CXCL10 in the parental A549 cells (Fig. 2E). In addition, STAT1 was also bound with the promoters of PD-L1 and CXCL10, particularly in 20 ng/mL of IFNα/γ-treated A549 cells (Fig. 2E). The results suggested that IRF-1 mediated intrinsic PD-L1 and CXCL10 expression and STAT1 particularly mediated IFNs-induced PD-L1 and CXCL10 expression in A549 cells.

### IFNα activates immune cells in vitro

**Fig. 3:**
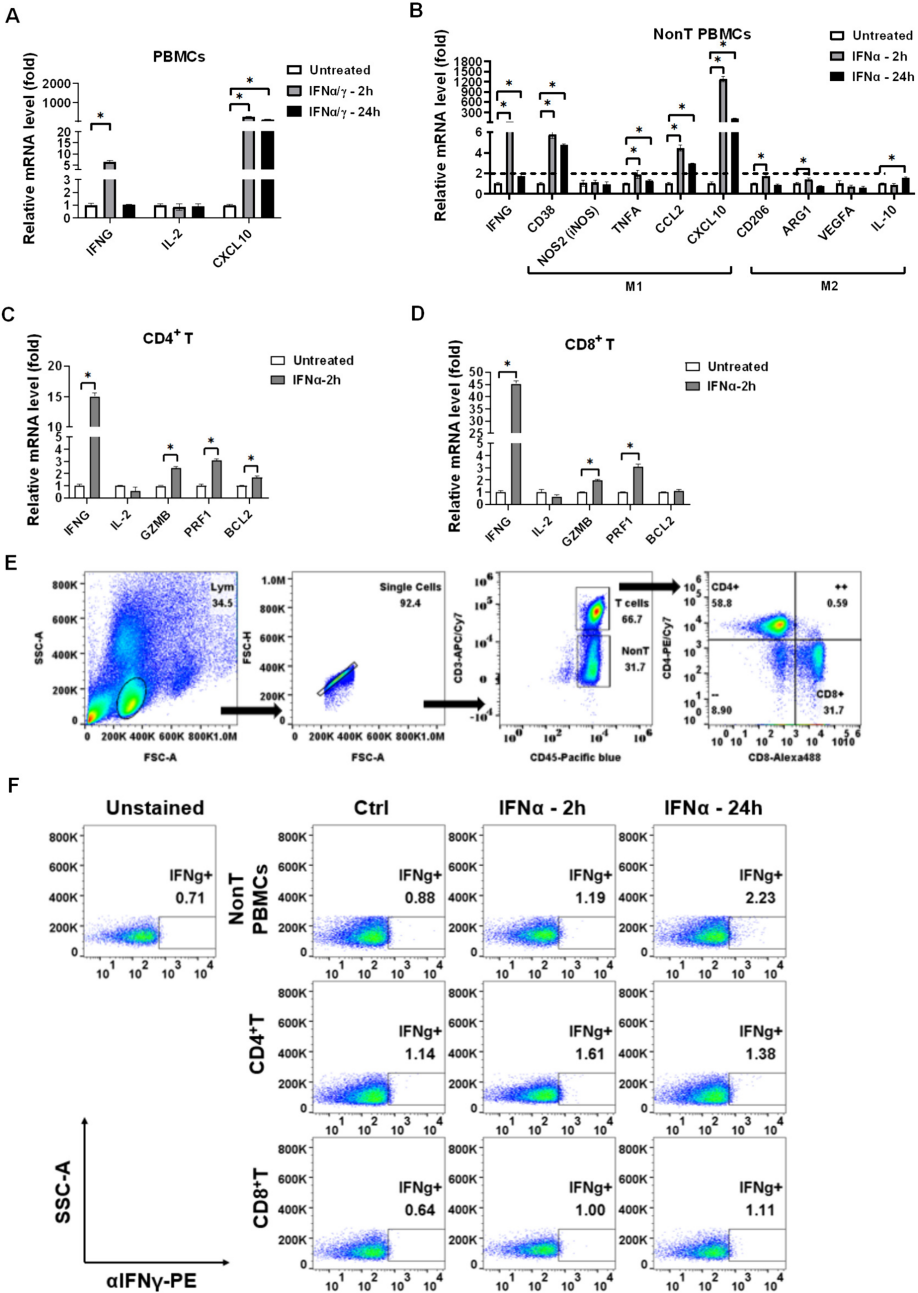
IFNα activates PBMCs and stimulates nonT PBMCs (monocytes) to express CXCL10. **A** qPCR was used to detect the activation marker IFNγ and IL-2 and chemokine CXCL10 in healthy PBMCs treated with 20 ng/mL of IFNα and IFNγ. **B** Immune activation marker IFNγ and M1 and M2 markers in nonT PBMCs (T lymphocytes were excluded) were investigated using qPCR after IFNα treatment for 2 h and 24 h. **C** The activation marker IFNγ, IL-2 and cytotoxic marker GZMB, PRF1, and anti-apoptosis marker BCL-2 were detected by qPCR in 20 ng/mL of IFNα-treated CD4^+^ T cells and **D** CD8^+^ T cells. **E** The healthy PBMCs were treated with 20 ng/mL of IFNα and analyzed by flow cytometry to **F** detect the IFNγ protein levels in nonT PBMCs, CD4^+^ T, and CD8^+^ T cells. CD4^+^ T and CD8^+^ T were gated by staining anti-CD45-Pacific blue, anti-CD3-APC/Cy7, anti-CD8-Alexa488, anti-CD4-PE/Cy7, and anti-IFNγ-PE.*p < 0.05

Besides regulations in tumors, IFNs are considered strong cytokines to activate immune cells. Therefore, we investigated whether IFNs activated the isolated PBMCs from a healthy volunteer, including nonT PBMCs (T cells are excluded), CD4^+^ T cells, and CD8^+^ T cells. We found that 20 ng/mL of IFNα/γ mixture induced activation marker IFNγ expression in the healthy PBMCs (Fig. 3A). Then, we validated that IFNα induced activation marker IFNγ expression in nonT PBMCs (Fig. 3B), whereas IFNα particularly induced M1 type markers, including CD38, CCL2, and CXCL10 rather than M2 type markers (Fig. 3B). The results also indicated that CXCL10 expression could not only be induced in IFNs-treated tumors but also in IFNs-treated monocytes. In addition, we validated that IFNα significantly increased activation marker IFNγ in CD4^+^ T cells (Fig. 3C) and CD8^+^ T cells (Fig. 3D). To validate the findings, flow cytometry was used to detect the protein levels of IFNγ in the IFNα-treated PBMCs (Fig. 3E), whereas IFNα increased IFNγ levels in nonT PBMCs, CD4^+^ T, and CD8^+^ T cells (Fig. 3F). The results revealed that IFNα potentially activated immune cells.

### CXCR3 abundantly expresses in T lymphocytes

**Fig. 4:**
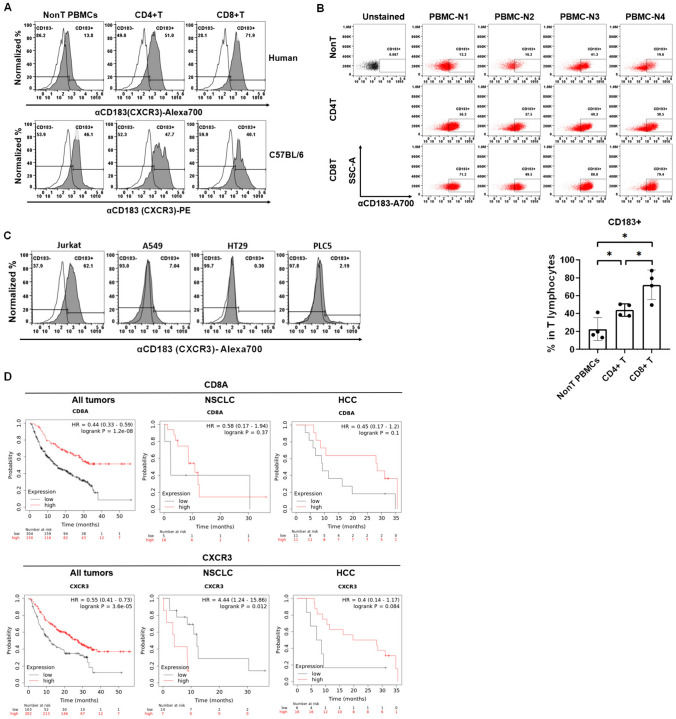
CXCR3 abundantly expresses in immune cells, particularly in human CD8^+^ T cells. **A** Flow cytometry was used to detect CXCR3 (CD183) expression in nonT PBMCs, CD4^+^ T, and CD8^+^ T cells from a healthy volunteer and a C57BL/6 mouse, whereas nonT PBMCs were residue of PBMCs after T lymphocytes isolation. Therefore, nonT PBMCs contain B cells, NK cells, and macrophages. CD4^+^ T and CD8^+^ T were gated by staining anti-CD3-APC/Cy7, anti-CD8-Alexa488, and anti-CXCR3-Alexa700 for human samples. Splenocytes were stained with anti-CD3-Pacific blue, anti-CD8-Alexa700, and anti-CXCR3-PE. **B** Moreover, healthy volunteers were enrolled in this study to validate the abundant CXCR3 expression in the isolated T lymphocytes (*n* = 4) analyzed by flow cytometry. **C** Meanwhile, CXCR3 expression was also investigated in the selected tumor cell lines, including lung cancer A549, colorectal cancer HT29, and liver cancer PLC5 cells by flow cytometry. T lymphoma Jurkat cells were used as a positive control. **D** Kaplan–Meier plotter was used to investigate the association among CD8A and CXCR3 levels with immunotherapeutic efficacy in patients with all cancers (*n* = 454), non-small cell lung cancer (NSCLC, *n* = 21), and hepatocellular carcinoma (HCC, *n* = 22).*p < 0.05

Next, the specific CXCL10 receptor CXCR3 (CD183) involved in NK and T lymphocyte migration was detected in the PBMCs and tumor cells to validate the abundant expression characteristic of CXCR3 in T lymphocytes. We found that CXCR3 expressed in the nonT PBMCs (including B, NK and, monocytes), CD4^+^ T, and CD8^+^ T cells in human PBMCs and mouse splenocytes (Fig. 4A). The CXCR3^+^ immune cell distribution was different between human PBMCs and mouse splenocytes, with higher CXCR3 in human T lymphocytes but similar distribution in mouse splenocytes (Fig. 4A). To validate the findings in human PBMCs, healthy volunteers were enrolled for detecting CXCR3 levels in the isolated PBMCs (*n* = 4). We validated that CXCR3 expression in CD8^+^ T cells was higher than in CD4^+^ T cells and nonT PBMCs (Fig. 4B). Therefore, CXCL10 may recruit T lymphocytes in the tumor microenvironment. To exclude CXCL10 function in tumor cells, the tumor cell lines including lung cancer A549, colorectal cancer HT29, and liver cancer PLC5 cells were selected to detect CXCR3 expression by flow cytometry compared to T lymphoma Jurkat cells, whereas Jurkat cells were used as a positive control. We, then, demonstrated that the selected tumor cells did not express CXCR3 (Fig. 4C). Even though there was no association with the small sample size of NSCLC (*n* = 21) and HCC (*n* = 22) (except for CXCR3 in NSCLC that CXCR3 resulted in poor survival rate in patients receiving immunotherapies, *p* = 0.012, Fig. 4D). We validated that high CD8A and CXCR3 levels were associated with a better survival rate in all cancer patients treated with immunotherapies (Fig. 4D).

### CT26 presents higher Ifnα/γ, Pd-l1, and Cxcl-10 levels compared to LL/2 to activate immune cells

**Fig. 5:**
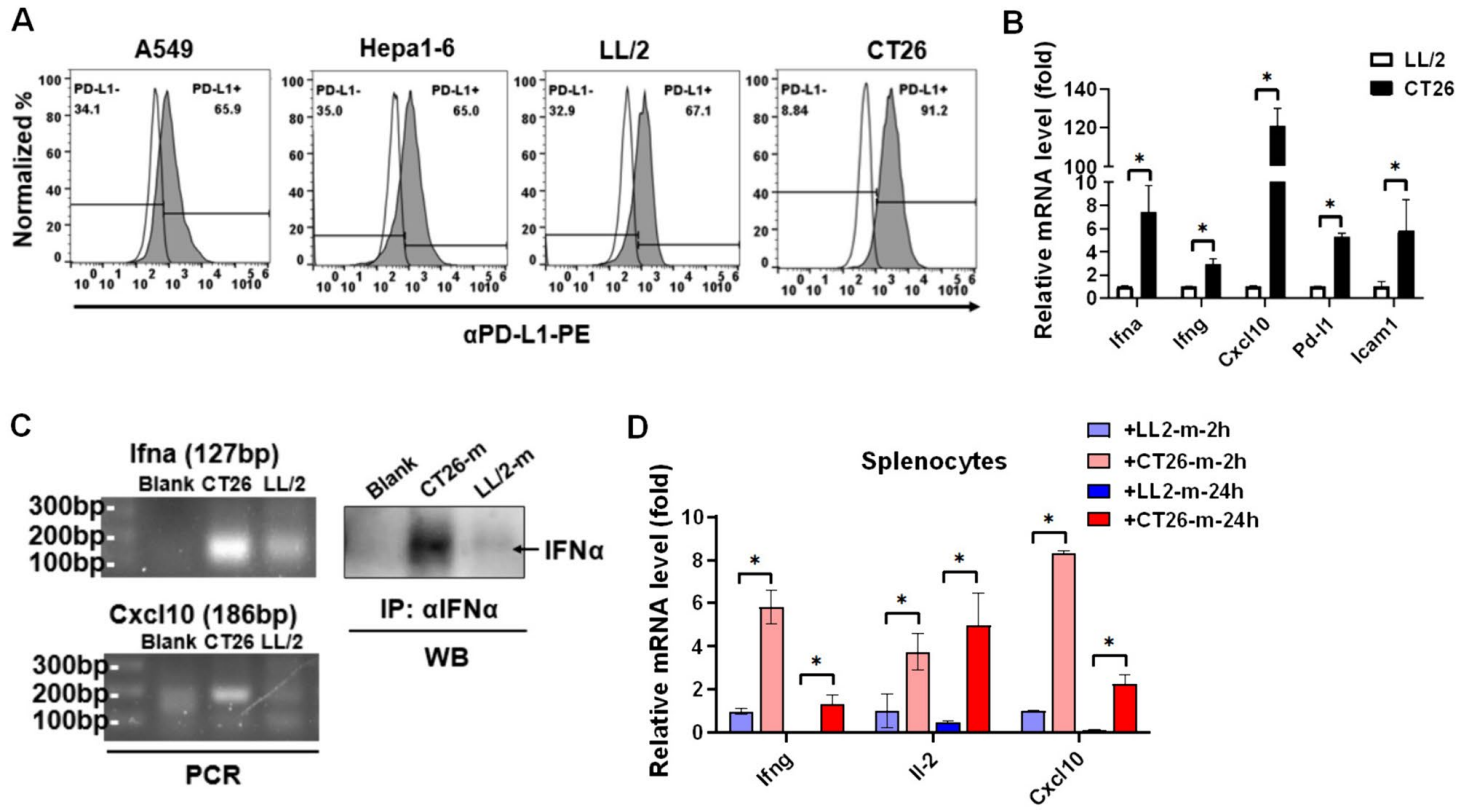
CT26 exhibits higher Ifna and Cxcl10 levels than LL/2 to potentially activate CD8^+^ T cells. A Flow cytometry was used to detect the PD-L1 expression in the selected tumor cell lines, including human lung cancer A549, mouse HCC Hepa1-6, mouse lung cancer LL/2, and mouse colon cancer CT26. **B** qPCR was used to compare the gene expression between LL/2 and CT26 cells. **C** PCR was used to validate the Ifna and Cxcl10 levels in LL/2 and CT26 cells. In addition, secreted IFNα in the LL/2 and CT26 medium (m) was captured by anti-IFNα antibodies with Dynabeads-Protein A and consequently detected using Western blots. **D** Activation markers Ifng, Il-2, and Cxcl10 were detected by qPCR in the mouse splenocytes treated with the culture medium (m) from LL/2 and CT-26. *p < 0.05

To validate that tumor-intrinsic IFNs and CXCL10 contribute to immunotherapeutic efficacy, the cell lines to induce syngeneic tumor mice were selected and investigated. Since CT26 exhibits αPD-(L)1 immunotherapy-sensitive but LL/2 exhibits immunotherapy-resistant [24, 25], we, therefore, selected CT26 and LL/2 for comparison and investigated the PD-L1 expression between the two cell lines. We found that PD-L1 was expressed in both LL/2 and CT26 but PD-L1 in CT26 (91.2%) was higher than that in LL/2 (67.1%, Fig. 5A). Moreover, other cell lines such as human lung cancer A549 and mouse HCC Hepa1–6 also exhibited similar levels of PD-L1 compared to LL/2 (65.9% in A549 and 65% in Hepa1–6, Fig. 5A). Therefore, LL/2 and CT26 were selected for further investigation and we analyzed additional gene expression between LL/2 and CT26. We found that Ifna, Ifng, Cxcl-10, Pd-l1, and Icam1 in CT26 were higher than that in LL/2 (Fig. 5B). To make sure the findings, PCR was used to detect Ifna and Cxcl-10, both overexpressed in CT26 cells (Fig. 5C). The results implied that IFNα secretion from CT26 (Fig. 5C) potentially activated immune cells. Therefore, the culture medium was collected from LL/2 and CT26 cells and treated with mouse splenocytes for 2 h and 24 h. We found that CD26 supernatant indeed significantly increased activation markers Ifng, Il-2, and Cxcl10 in mouse splenocytes compared to the LL/2 supernatant (Fig. 5D).

**Fig. 6:**
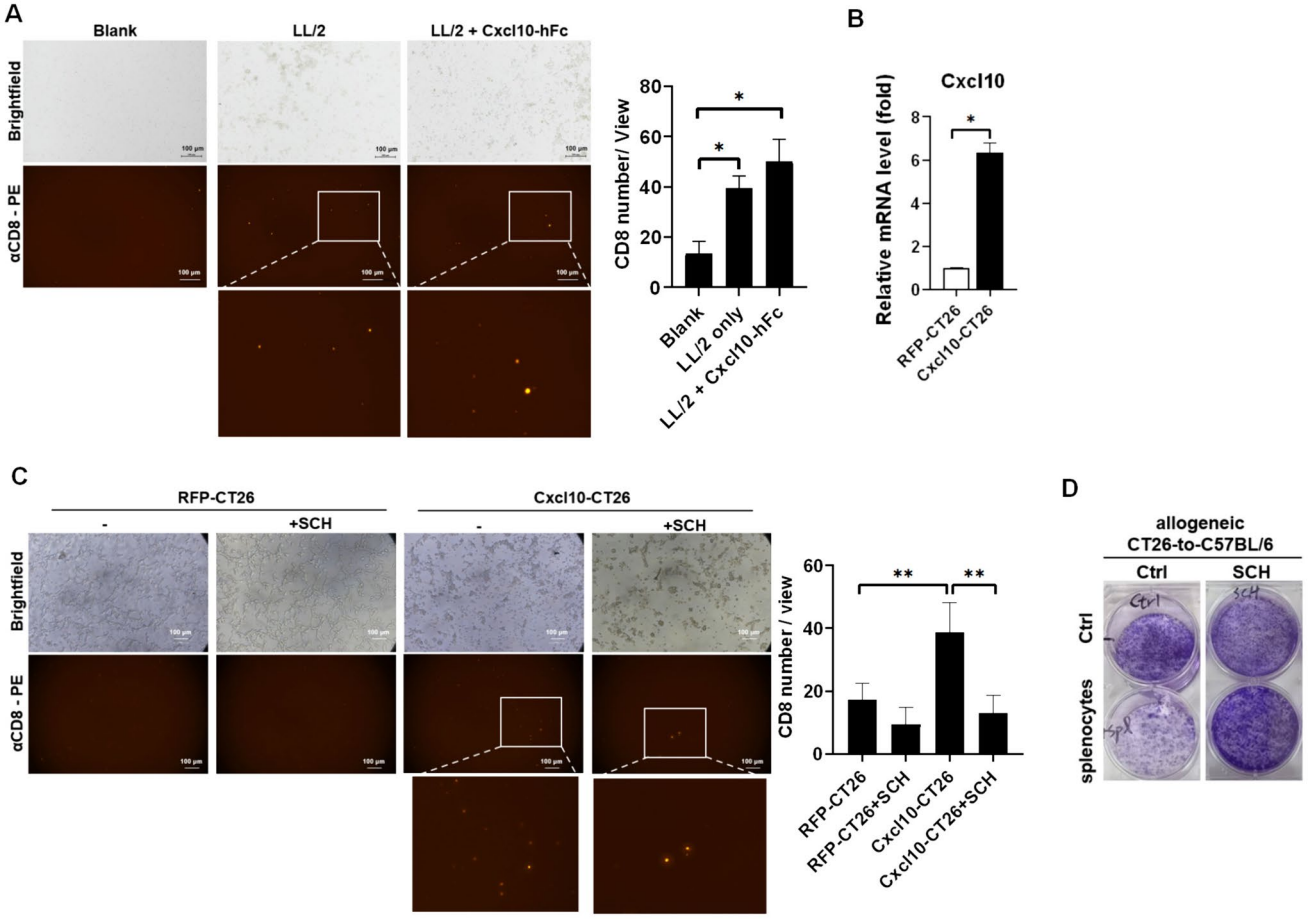
CXCR3 contributes to T cell migration and activation. **A** A transwell assay was used to determine the CD8^+^ T cell migration in 20 ng/mL of Cxcl10-hFc treatment for 24 h. Mouse splenocytes was pre-labeled with anti-CD8-PE and loaded on the up chamber and LL/2 was cultured in a 24-well plate. Each group contain *n* = 3 for statistical analysis. **B** Cxcl10 was overexpressed and validated in CT26 using qPCR, **C** which was cultured in a 24-well plate with mouse splenocytes pre-labeled with anti-CD8-PE and loaded on the up chamber for 24 h to validate the Cxcl10 function for CD8^+^ T cell migration. Each group contains at least *n* = 3 for statistical analysis. **D** Colony formation was used to investigate the anti-tumor effect in an allogeneic CT26-to-C57BL/6 splenocytes model. 100 nM of SCH546738 (SCH), an inhibitor of CXCR3, was used to block T cell migration and activity. *p < 0.05, **p < 0.01

Our results indicated that IFNs mediated CXCL10 expression in tumor cells (Fig. 2). The CT26 supernatant also contains CXCL10, which has the potential to recruit T lymphocytes, transferring the “cold tumor” to the “hot tumor” characteristic. Therefore, we constructed and purified mouse Cxcl10 conjugated with human IgG2 Fc (Cxcl10-hFc) and overexpressed Cxcl10 in CT26 to validate the role of CXCL10 in CD8^+^ T cell recruitment in vitro. We demonstrated that Cxcl10-hFc significantly enhanced CD8^+^ T cell migration in vitro (Fig. 6A). Moreover, Cxcl10-overexpressed CT26 (Fig. 6B) significantly recruited CD8^+^ T cells in vitro, whereas 100 nM of SCH549738, an inhibitor of CXCR3, inhibited Cxcl10-CT26-mediated CD8^+^ T cell migration effect (Fig. 6C). Meanwhile, we found that allogeneic C57BL/6 splenocytes suppressed CT26 colony formation, and treatment with 100 nM of SCH549738 inhibited the allogeneic splenocytes-mediated anti-CT26 effect (Fig. 6D). These results revealed that CXCL10 mediated T cell recruitment and CXCR3 may be involved in anti-tumor activity.

### CT26-derived tumor sensitizes to αPD-L1scFv-hFc immunotherapy but LL/2-tumor resists

**Fig. 7:**
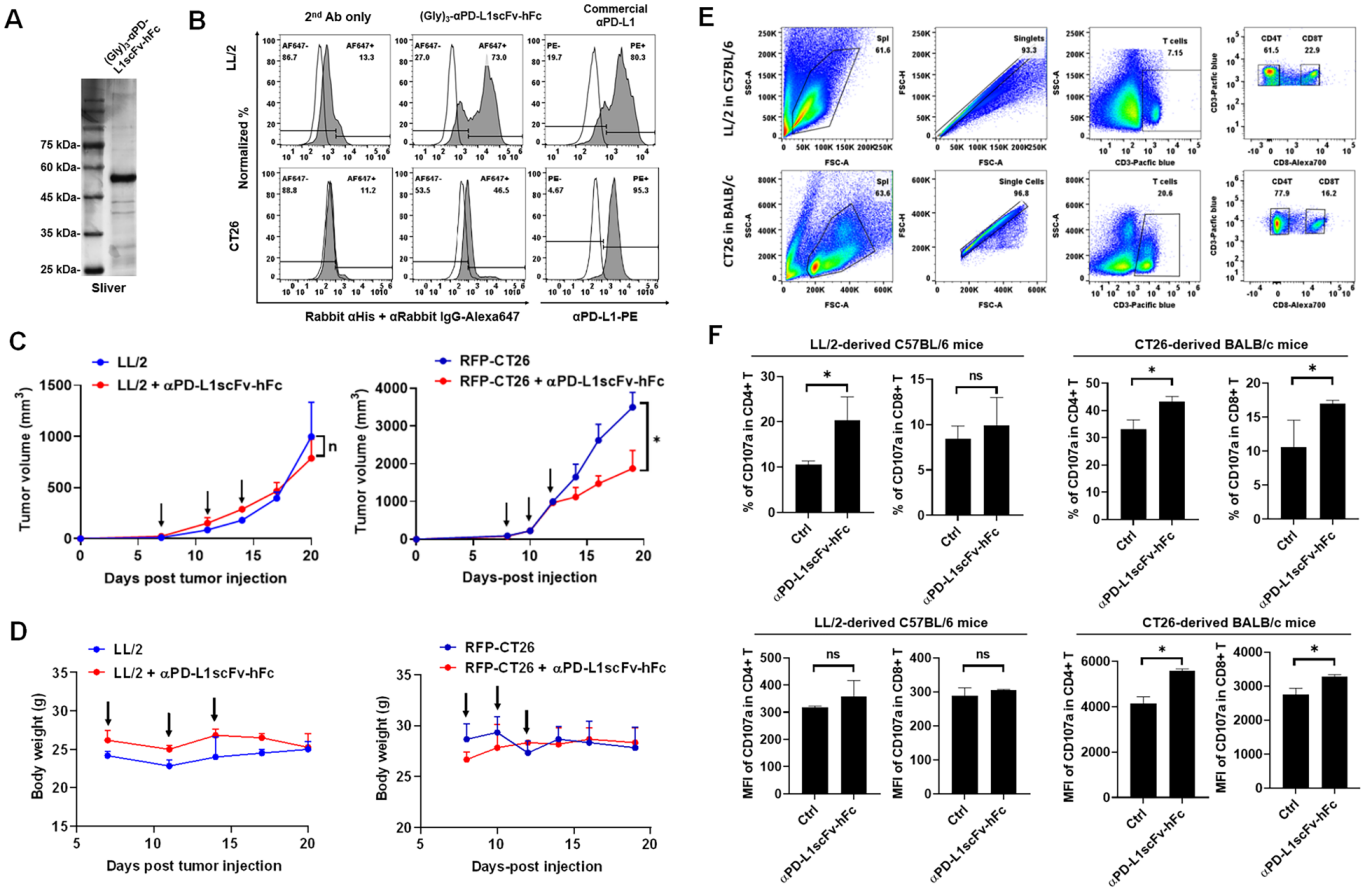
CT26 exhibits αPD-L1scFv-hFc immunotherapy-sensitive but LL/2 exhibits resistant. **A** αPD-L1scFv-h(human)Fc was constructed and purified by Ni-column and detected using silver staining. **B** Flow cytometry was used to detect the binding capacity of αPD-L1scFv-hFc with LL/2 and CT26 cells in vitro, whereas a commercial αPD-L1 was used as a positive control. **C** The LL/2 and CT26 cells were subcutaneously injected into their syngeneic mice, BALB/c (*n* = 3) and C57BL/6 (*n* = 3), respectively. Individual 100 μg of αPD-L1scFv-hFc was administrated intraperitoneally on day 7, 11, and 14 for LL/2-derived mice and on day 8, 10, and 12 for CT26-derived mice (indicated by arrows). **D** Body weights were documented to be used as a toxicity marker in αPD-L1scFv-hFc administration. **E** Splenocytes harvested after the end of tumor size measurement from LL/2-derived C57BL/6 (*n* = 3) and CT26-derived BALB/c mice (*n* = 3) were analyzed using flow cytometry to **F** detect the activation marker CD107a expression in T lymphocytes, including CD4^+^ T and CD8^+^ T cells. *p < 0.05

To investigate the immunotherapeutic efficacy in the selected syngeneic tumor mice, we constructed and purified αPD-L1scFv-hFc antibody based on Atezolizumab, an antibody targeting PD-L1, which was detected by silver staining (Fig. 7A). We validated that αPD-L1scFv-hFc was capable of binding to LL/2 and CT26 cells in vitro detected using flow cytometry, whereas a commercial anti-PD-L1 antibody was used as positive control (Fig. 7B). We noticed that αPD-L1scFv-hFc interacted with LL/2 stronger than CT26, whereas a commercial αPD-L1 staining still showed PD-L1 in CT26 was higher than that in LL/2 (Fig. 7B). Then, we still found that LL/2 was resistant but CT26 was sensitive to αPD-L1scFv-hFc immunotherapy in the syngeneic tumor mice, whereas tumor inhibition appeared after three times αPD-L1scFv-hFc intraperitoneal injection (Fig. 7C). Administration of αPD-L1scFv-hFc did not cause apparent toxicity by measuring the mouse body weight in LL/2-derived C57BL/6 and CT26-derived BALB/c mice (Fig. 7D). The splenocytes were harvested and investigated for detecting the expression of activation marker CD107a in T lymphocytes using flow cytometry (Fig. 7E). We noticed that total T lymphocytes in splenocytes of CT26-derived BALB/c mice (20.6%) were higher than that in LL/2-derived C57BL/6 (7.15%, Fig. 7E). We further demonstrated that αPD-L1scFv-hFc significantly increased activation marker CD107a in CD4^+^ T and CD8^+^ T cells in CT26-derived tumor mice (Fig. 7F). The results revealed that αPD-L1scFv-hFc immunotherapy stimulated and activated T lymphocytes in CT26-derived BALB/c immunotherapy-sensitive model that may be the “hot tumor” characteristic with higher T cell population and tumor-intrinsic IFNs and CXCL10 expression.

## Discussion

Our study reveals the critical role of tumor-intrinsic IFNs and CXCL10 in determining the potential efficacy of anti-PD-(L)1 immunotherapy. The findings suggest that IFNs-mediated CXCL10 expression, along with PD-L1, plays a significant role in modulating the immune response within the tumor microenvironment. Therefore, CXCL10 and PD-L1 both were associated with a survival rate in all patients treated with immunotherapies. While PD-L1 is not uniformly overexpressed across all tumors, CXCL10 consistently exhibits elevated levels in tumors, particularly in lung, colon, and liver cancers, as a reliable biomarker for predicting immunotherapeutic efficacy [26]. Some tumor treatments to induce CXCL9 and CXCL10 expression in tumor cells also potentially benefit tumor immunotherapeutic efficacy when administered by a combination strategy [27, 28].

Current findings suggest that PD-L1 levels are associated with the anti-tumor efficacy of immunotherapies by targeting PD-(L)1 [29]. Tumor cells, including LL/2 and CT26, exhibit high PD-L1 levels, 67.1% in LL/2 and 91.2% in CT26 (Fig. 5A), which present opposite responses to αPD-L1 immunotherapy, immunotherapy-sensitive in CT26 and immunotherapy-resistant in LL/2 (Fig. 7C). The results may indicate that PD-L1 expression in tumors is essential but not critical for determining αPD-L1 immunotherapy. To investigate the detailed mechanism, we proposed that a “hot tumor” with higher T cell infiltration is the major reason to benefit αPD-(L)1 immunotherapies. Therefore, CXCL10 function for recruiting T lymphocytes is analyzed in comparison with PD-L1 in this study. We investigated PD-L1 and CXCL10 levels and correlation and found that PD-L1 and CXCL10 shared the same IFNs-mediated signaling pathways in tumor cells (Fig. 2), particularly both were associated with a better survival rate in tumor patients receiving immunotherapies (Fig. 1D). Therefore, PD-L1 expression is essential, but not enough for αPD-(L)1 immunotherapies. “Hot tumor” characteristic is critical that tumor-intrinsic IFNs and CXCL10 in CT26 cells potentially contributed to the “hot tumor” characteristic by recruiting T lymphocytes to the tumor microenvironment.”

According to previous literature, in the several syngeneic tumor models for testing the αPD-L1 immunotherapy, including the tumors derived by CT26, RENCA, 4T1, B16F10 AP3, LL/2, and MC38, there is only CT26 sensitized to αPD-L1 immunotherapy [24]. Therefore, we further investigate and demonstrate the detailed mechanism in this study by comparing the tumor-intrinsic IFNs and CXCL10 levels in the immunotherapy-sensitive CT26 and immunotherapy-resistant LL/2. Compared to LL/2, we found that CT26 exhibited higher IFNs and CXCL10 levels in this study (Fig. 5B), which potentially further increased T lymphocyte recruitment in tumor microenvironment. Particularly, the total T lymphocytes in splenocytes of CT26-derived BALB/c mice (20.6%) were higher than that in LL/2-derived C57BL/6 (7.15%, Fig. 7E). In addition, Suzanne I.S. Mosely and colleagues have demonstrated that NK (26.92%), CD4^+^ T (7.24%), and CD8^+^ T (5.04%) are indeed higher in immunotherapy-sensitive CT26-derived tumor models compared to others, including LL/2 [24]. Besides T lymphocytes, NK cells also present high CXCR3 expression for cell migration [30]. CT26 may also recruit NK cells to suppress tumor cell proliferation in the CD26-derived tumor mice that needs further investigation furthermore.

**Fig. 8:**
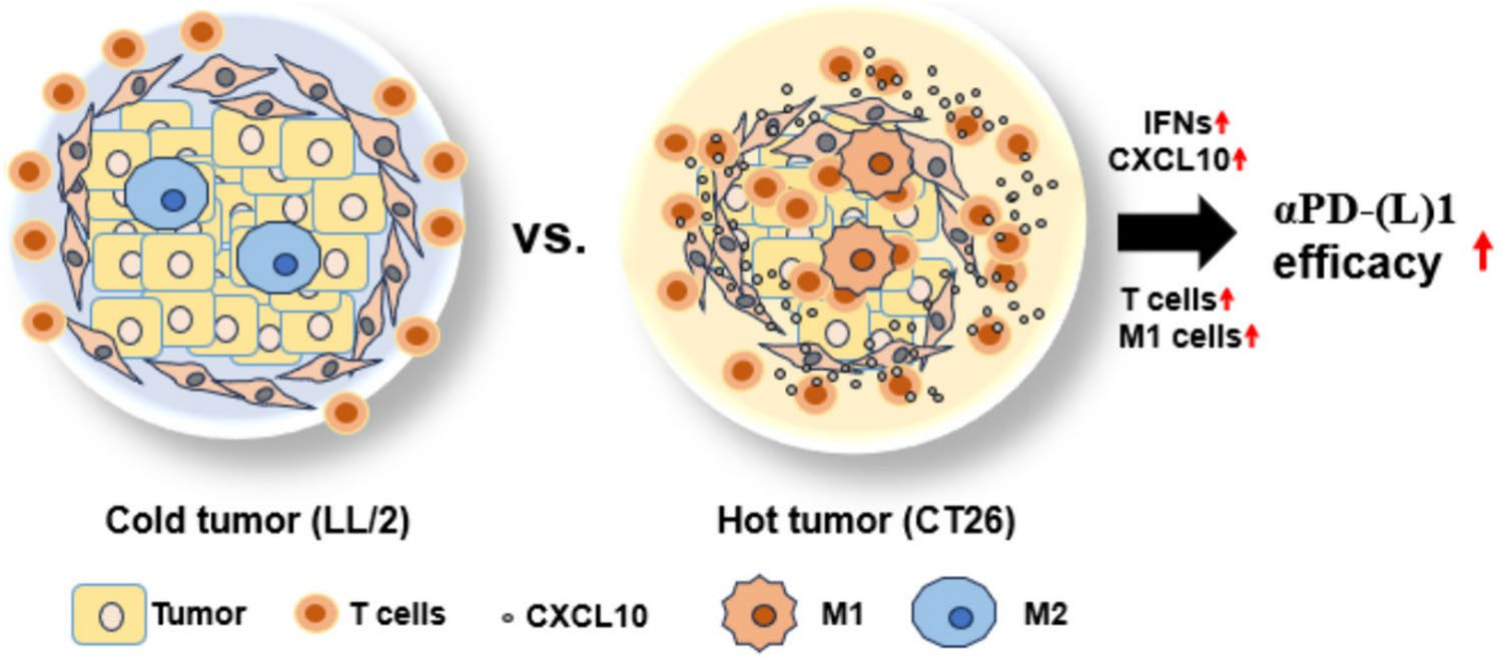
The schematic diagram illustrates the mechanism for improving αPD-(L)1 immunotherapeutic efficacy. CT26 exhibits higher IFNs, CXCL10, T cell infiltration, and M1 differentiation called “hot tumor” compared to LL/2 “cold tumor,” which potentially results in better αPD-(L)1 immunotherapeutic efficacy

Our analysis also revealed a potential mechanistic link between immunotherapy-sensitive CT26 cells and the activation of CD8^+^ T cells. The comparison between LL/2 and CT26 cell lines, representing immunotherapy-resistant and immunotherapy-sensitive tumors, respectively [24, 25], further supports the roles of IFNα and CXCL10 in mediating anti-tumor immunity. CT26 cells exhibited higher expression levels of IFNs and CXCL10 compared to LL/2 cells (Fig. 5B), highlighting the association between these cytokines and immunotherapy responsiveness. Our findings also indicate that IFNα and CXCL10 recruit and activate CD8^+^ T cells toward the tumor cells (Fig. 6A–C), potentially transforming “cold tumors” into “hot tumors” that are more responsive to immunotherapy (Fig. 8).

In addition to tumor-intrinsic CXCL10 expression, it is noteworthy that tumor-intrinsic IFNα may also contribute to M1 polarization (Fig. 3B), leading to M1 macrophage-mediated CXCL10 overexpression (Fig. 3B). This phenomenon is particularly important as it facilitates the recruitment and activation of CD8^+^ T cells, preventing the function of immune-suppressing M2 macrophages which typically predominate in the tumor microenvironment [31]. The polarization of macrophages toward the M1 phenotype promotes an immune-stimulatory microenvironment conducive to anti-tumor immunity [32]. Therefore, the observed upregulation of CXCL10, driven by tumor-intrinsic IFNα and potentially mediated by M1 polarization, likely plays a crucial role in facilitating the recruitment and activation of CD8^+^ T cells, thereby enhancing the efficacy of immunotherapeutic interventions targeting PD-(L)1.

Our findings also highlight the critical role of CXCR3 in the recruitment and activation of CD8^+^ T cells within the tumor microenvironment and its potential implications for immunotherapeutic efficacy. CXCR3 is a surface receptor expressed on T lymphocytes that responds to chemokines such as CXCL9 and CXCL10 [27]. CXCR3 activation mediates the interaction between CD8^+^ T LFA-1 and tumor ICAM-1 [33, 34], further promoting T cell adhesion and activation within the tumor microenvironment [23]. As a result, targeting CXCR3 signaling pathways by CXCL9/10 could be a promising anti-tumor strategy [35].

## Conclusion

Overall, our study provides compelling evidence for the pivotal role of tumor-intrinsic IFNα and CXCL10 in orchestrating the immunotherapeutic response within the tumor microenvironment. IFNα specifically mediated CXCL10 expression in both tumor cells and PBMCs to recruit and activate CD8^+^ T cells, which expresses CXCR3 abundantly. Therefore, tumor-intrinsic IFNα and CXCL10 potentially transfer “cold tumor” to “hot tumor,” to be helpful for anti-PD-(L)1 immunotherapies (Fig. 8). Furthermore, administration of additional IFNα and CXCL10 may be a candidate tumor treatment for CD8^+^ T cell-specific recruitment to enhance the efficacy of anti-PD-(L)1 immunotherapy and improve clinical outcomes for patients with various cancers.

## Data Availability

All data generated or analyzed during this study are included in this published article. The data analyzed in the current study are available upon reasonable request.

## Abbreviations

ATCC: American Type Culture Collection
CCL5: Chemokine (C–C motif) ligand 5
ChIP: Chromatin immunoprecipitation
COAD: Colon adenocarcinoma
CTLs: Cytotoxic T lymphocytes
CXCL10: C-X-C motif chemokine ligand 10
DCs: Dendritic cells
EGA: European Genome-phenome Archive
GEO: Gene Expression Omnibus
GEPIA: Gene Expression Profiling Interactive Analysis
HCC: Hepatocellular carcinoma
IFNα: Interferon α
IFNγ: Interferon γ
IRF-1: Interferon regulatory factor 1
IPTG: Isopropyl-β-d-thiogalactopyronoside
LIHC: Liver hepatocellular carcinoma
LUAD: Lung adenocarcinoma
NK: Natural killer
NSCLC: Non-small cell lung cancer
PBMCs: Peripheral blood mononuclear cells
PD-L1: Programmed death-ligand 1
qPCR: Quantitative polymerase chain reaction
RBC: Red blood cells
RT: Radiotherapy
STAT1: Signal transducer and activator of transcription 1
TCGA: The Cancer Genome Atlas
